# Good role models? Tooth brushing capabilities of parents: a video observation study

**DOI:** 10.1186/s12903-021-01823-6

**Published:** 2021-09-24

**Authors:** Renate Deinzer, Sadhvi Shankar-Subramanian, Alexander Ritsert, Stefanie Ebel, Bernd Wöstmann, Jutta Margraf-Stiksrud, Zdenka Eidenhardt

**Affiliations:** 1 Department of Medicine, Justus-Liebig-University Giessen, Klinikstr. 29, 35392 Giessen, Germany; 2 Department of Medicine, Justus-Liebig-University Giessen, Schlangenzahl 14, 35392 Giessen, Germany; 3 Marburg, Germany; 4 Department of Medicine, Institute of Medical Psychology, Justus-Liebig-University Giessen, Klinikstr. 29, 35392 Giessen, Germany

**Keywords:** Oral health, Oral hygiene, Tooth brushing, Dental plaque, Health behavior, Health education, Dental, Community dentistry, Parents, Behavior observation techniques

## Abstract

**Background:**

Research in adolescents reveals that they are not capable to remove dental plaque effectively. Inconsistent application of brushing techniques and neglect of certain areas while brushing are very common. As parents play a major role in the oral health education, the present study aimed to examine and describe the tooth brushing performance of the parents of adolescents.

**Methods:**

Parents of adolescents (N = 66) were asked to perform oral hygiene to the best of their capabilities in front of a video camera and dental plaque was recorded before and afterwards. Papillary bleeding was also assessed.

**Results:**

The tooth contact time (i.e. net brushing duration) averaged 155 s ± 58 s. Gingival margins showed persistent plaque at 68% ± 14% of the sections assessed. Papillary bleeding was found at 52% ± 18% of the papillae. Parents brushed inner surfaces lesser than the outer surfaces (41 s ± 24 s vs. 73 s ± 33 s) and 29% of the parents completely missed the inner surfaces of at least one sextant. On the outer surfaces parents predominantly applied circular movements (66% ± 25% of brushing time). Vertical brushing prevailed on the inner surfaces (52% ± 30%). However, horizontal scrubbing was also very common (46% ± 31%).

**Conclusions:**

Parents’ tooth brushing performance was neither effective in terms of plaque removal nor did they fully comply with tooth brushing recommendations such as considering all inner surfaces when brushing or application of other than horizontal movements to lateral surfaces. Regarding oral hygiene, parents should not only be a good role model in terms of timing, frequency and duration of tooth brushing but should also be able to demonstrate how to brush teeth completely and effectively. The current research indicates that they might lack the latter skill.

**Supplementary Information:**

The online version contains supplementary material available at 10.1186/s12903-021-01823-6.

## Background

Persistent marginal plaque is an important etiological agent of periodontal diseases. Tooth brushing is the most appropriate measure to remove plaque and maintain oral health [1, 2]. In line with this, several epidemiological studies reveal that the self-reported oral hygiene behaviour corresponds with the general recommendations by the dental professionals. This is in reference to the frequency and duration of tooth brushing. Yet, the prevalence of gingivitis and periodontitis is high in Germany and worldwide [3–6]. Hence, it is very likely that the efficacy of tooth brushing tends to be low or insufficient despite the regular performance of oral hygiene. This assumption is supported by many studies which report persistent plaque after brushing in different age groups [7–14].

The above finding led to a series of video observation studies in adolescents (10–19 years) and young adults (20–30 years). These observational studies aimed at understanding the origin of the deficits by a thorough observation of what people exactly do when they are brushing their teeth [14–16]. Results showed that adolescents and young adults brushed their teeth for an average of more than three minutes, at least when they were asked to brush as thoroughly as possible. However, the brushing pattern was rather unsystematic. There was a striking neglect of the inner surfaces. Furthermore, they applied brushing techniques inconsistently and scrubbing was one of the most prominent brushing movements on the lateral surfaces [14, 15, 17, 18]. These behaviours considerably deviated from recommendations given in group and individual prophylaxis programs [19, 20]. Such programs have been mandated by social legislation since 1989 from kindergarten until the age of 18 in order to enable all children to develop proper oral hygiene behaviours [16]. The programs comprise of either one or two instructions per year and thus strongly rely on parental support by continuous demonstration and training of effective oral hygiene behaviour at home. However, if the parents show similar inappropriate tooth brushing as observed in adolescents and young adults [14–17] the support they can give might be limited. Therefore, it may be worthwhile to assess the tooth brushing skills of parents. This is also interesting from a second point of view; parents of adolescents typically are between 30 and 50 years old. This is the age when the first signs of periodontitis are usually detected [4, 5]. Therefore, it is very important to gain insights into the tooth brushing behaviour of the adults in this age group.

Thus, the major aim of the present study was to examine and describe the tooth brushing performance of the parents of adolescents.

## Methods

### Ethics

This study was conducted in accordance with the Declaration of Helsinki and ethical permissions were granted by the Ethics Board of the Medical Faculty at the University of Giessen, Germany (No: 255/18). All participants provided written informed consent.

### Participants and recruitment

The data presented here are part of a large cross-sectional study that sought to investigate the oral hygiene behaviour of adolescents and their parents [see 17]. The study was conducted at the Institute for Medical Psychology, Justus Liebig University in Giessen from August to December 2019. The participants were recruited in pairs (the adolescent with that parent who ascribed himself or herself the main responsible for the oral hygiene education in the adolescent’s childhood) via social media advertisements, emails, letters and flyers distributed in the schools. The prospective participants expressed their interests by a response to the advert. Thereafter, they were thoroughly informed about this study through a telephonic conversation. An appointment was made after taking into account the following inclusion and exclusion criteria: *Inclusion Criteria*: (1) Only parents of adolescents who had been enrolled in kindergarten in Germany (important for analyses of the adolescents); (2) age of the adolescents is 10 or 15 years (± 12 months)^1^; (3) Predominant use of a manual toothbrush (at least two thirds of all brushing events); (4) Very versatile in German language as the questionnaires and the entire study was designed in German; (5) Presence of more than 20 natural teeth. *Exclusion criteria*: (1) Cognitive or physical impairment that affects tooth brushing; (2) Pregnancy/ lactation; (3) Fixed orthodontic appliances; (4) Removable prosthesis or dentures; (5) Oral piercings/ Dental jewelry; (6) Dental prophylaxis in the last four months; (7) Antibiotic consumption in the past three months; (8) Previous training of the parents in any of the dental professions.

Participants were given 50 Euros as an incentive for taking part in this research work. Parent and adolescent pairs were examined at the same time in separate examination rooms.

The current analysis focuses on the oral hygiene behaviour of the parents. A detailed description of the behaviour of the adolescents is provided by Eidenhardt et al. [17]. Initially, the authors of this study had planned to include 100 adolescents with their parents. However, assessments had to be terminated prematurely after 66 parents-adolescent pairs due to two unforeseeable situations. Firstly, there was a serious cyber-attack in early December 2019 at the Justus Liebig University in Giessen, which led to a complete shutdown of the internal networks including computers until February 2020. Secondly, in March 2020 the pandemic COVID-19 led to a nation-wide lockdown in Germany, making further recruitment and assessment impossible. At that time, a suitable date for further assessments was not foreseeable. In addition, the pandemic caused people to become concerned about hygiene. The researchers perceived that this might affect participation and the tooth brushing behaviour.

### Procedures

Participants were instructed to refrain from any oral hygiene procedures at least 4 h before the scheduled appointment. Before examination, the respective parents were randomly assigned to one of the examiners in this study (ZE, DB or MS) and a dentist (AR, PH, or TS).

To provide a comprehensive overview, the design of the complete cross-sectional study is described in this paper. However, the present analysis focuses the oral hygiene behaviour of the parents only. The examiner welcomed parent-adolescent pairs in a first room, informed them about the study procedures and obtained their written consent to participate. The parent and their child were then seated in two separate rooms to fill out some questionnaires (not relevant for the current analyses). Thereafter, plaque and papillary bleeding were assessed in one of the dental examination rooms. The examiner then brought the participant to another room equipped with a washbasin and a tablet computer which served both as a camera and a mirror. A red transparent sheet was used to cover the surface of the tablet computer in order to make the plaque staining (Mira 2 tone plaque disclosing agent; Miradent, Germany) applied during the first dental examination invisible to the participants. Three further side cameras served as a supplement, when the tablet computer failed to record the tooth brushing performance properly. A standard toothbrush (Elmex InterX Kurzkopf medium, GABA GmbH) and toothpaste (Elmex; GABA GmbH) were provided. Further, interdental cleaning aids such as dental floss (waxed and unwaxed; Elmex; GABA, GmbH), super floss (Meridol; GABA GmbH), interdental brushes (Elmex: size 2 and 4, GABA GmbH) and interdental sticks (Tepe; Tepe D-A-C-H GmbH) were provided. The participants were instructed that they could use any or all of the cleaning devices and were left alone after that. The examiner then instructed from an adjacent room through a microphone to clean their teeth to the best of their abilities and recorded their oral hygiene performance. Participants communicated via a ceiling microphone when they had finished their tooth brushing. The examiner then accompanied them to the dentist who assessed the dental plaque after brushing, the dental status and the periodontal status after which the participants filled out a second questionnaire (also not relevant for the present analyses).

### Observed oral hygiene behaviour

#### Observation procedures

Four independent examiners analysed the videos of tooth brushing (SS, SE, AR, and KB). Three were blinded for the clinical status of the participants. One of the examiners (AR) had performed some of the dental examinations. This bias was reduced by sufficient time gap of around 12–13 weeks between the clinical assessment (August–December 2019) and the video analysis (April–May 2020). The analyses were done by means of the observational software Mangold Interact 18 (Mangold International GmbH, Arnstorf, Germany) with methods that had been established earlier [15, 16].

#### Behavioural parameters

Parents’ tooth brushing behaviour was examined with regard to the same parameters used to describe their children’s behaviour [17]. These were (1) Tooth contact time (time when the toothbrush touches the teeth without any interruptions like spitting, rinsing etc.). Within tooth contact time: (2) time of tooth contact at occlusal, inner or outer surfaces, respectively; (3) time of tooth contact per sextant (for the inner and outer surfaces) or quadrant (for occlusal surfaces), respectively; (4) time of tooth contact at outer surfaces when the mandible was closed (brushing in the so-called tiger bite); (5) time of brushing movements at lateral surfaces as either horizontal, vertical, circular, Modified Bass Technique or no brushing movement at all. Brushing movements were not coded at occlusal surfaces, as in general no movements other than horizontal movements were seen on these surfaces in previous studies.

For the analyses of the parents’ children, benchmarks were defined. These benchmarks have their origin in the recommendations they are given during oral hygiene education in kindergarten and school [17]. The same benchmarks were applied here to describe their parents’ behaviour. They are derived from the publicly available tooth brushing song used for teaching the children [19]. The benchmarks are [17]: Total tooth contact time of at least 97.5 s; occlusal surfaces brushed for at least 7.5 s per quadrant; outer surfaces brushed with circling movements; outer surfaces brushed two antagonistic sextants at a time while the jaws are closed (so-called tiger bite); each double sextant of outer surfaces brushed for at least 7.5 s; inner surfaces brushed with vertical movements; each inner sextant brushed for at least 7.5 s.

Furthermore, an overall quality index for the distribution of brushing time across surfaces and sextants (QIT-S) developed by Deinzer et al. [15] was computed from the data assessed for inner and outer surfaces respectively. Scores 0–5 indicate that 0–5 sextants were brushed for at least 1 s (brushing of less than a second is considered as neglect of the respective sextant). Score 6 indicates that every sextant was brushed for at least 1 s but less than 3.5 s, while score 7 and 8 indicate brushing of 3.5–5 s and 5–7.5 s, respectively. Score 9 is given when all sextants were brushed for at least 7.5 s.

#### Calibration of observers

For calibration, the examiners first got a written and oral instruction by an experienced examiner (SE) describing exactly the behavioural categories and the use of the observation software. Then, examiners analysed videos that were from previous studies, where the above-mentioned parameters already had been coded by calibrated examiners [13, 16]. Tooth contact time and surfaces were calibrated with five different videos. The other parameters (sextants and quadrants, brushing movements) were more intricate and hence ten different videos were used to calibrate. It was considered a successful calibration when the intraclass correlations (ICCs) were ≥ 0.9. All examiners fulfilled the criterion.

As the entire observation was dependent on the tooth contact time, this parameter was double coded by two calibrated examiners (MS and SS). Both observers agreed very accurately *(ICCs > 0.999)*. The other parameters were observed by one examiner (Surfaces: SE; Sextants and quadrants: AR; brushing movements: SS). To ensure reliability of these observations 10 randomly chosen videos were double coded by another calibrated examiner (surfaces: WP; sextants/ quadrants: TS; movements: KB) who was blinded to the analyses of other examiners. ICCs ≥ 0.80 indicate a fair interrater reliability. By these double coding an observation drift of the observer of the movements (SS) became obvious. The phenomenon of observer drift may occur over time, especially in lengthy research studies. The observers become inconsistent in the criteria they use to make their record and observations. Thus, KB re-analyzed all videos regarding brushing movements and WP did the double coding of 10 videos. The resulting ICCs of the final data were above 0.86 for sextants 1, 2, 5 and above 0.91 for all other parameters besides circular and vertical movements. The brushing movements within the video of one participant were very ambiguous. This led to ICC values of 0.77 and 0.64 for circular and vertical movements, respectively. However, when this video was excluded from ICC analyses, the ICCs of the remaining 9 videos with regard to circular and vertical movements were 0.99 and 0.96 respectively. This person also showed outlying values (see below) and was thus excluded from further analyses.

### Clinical data

The dentists (AR, PH and TS) were calibrated before the study started by an experienced dentist (WP) who was not involved in this study. A successful calibration was considered when more than 90% of the scorings were identical and less than 10% never deviated by more than 1 score in five subsequent patients. This procedure was similar to previous studies [14, 15]. All the dentists were blinded with respect to the oral hygiene behaviour and the questionnaire data of the parents. Plaque had been stained with a two-tone plaque disclosing agent (Miradent Mira-2-Ton®; Miradent, Germany). All clinical parameters were assessed on the existing teeth including the third molars. However, for ease of comparison to representative surveys, the DMFT is calculated without the inclusion of the 3rd molars according to the principles of the World Health Organization [21].

The following clinical parameters were recorded:

1. Dental status (healthy teeth, decayed, filled and missing teeth; teeth with partial and full crowns; pontics) and Papillary bleeding index (PBI, Saxer and Mühlemann [22] as modified by Rateitschak et al. [23]). The PBI was assessed at all inner and outer surfaces. Each of the surfaces was given a score from 0 to 4 (0 = no bleeding; 1 = single bleeding point; 2 = several bleeding points or thin line; 3 = interdental triangle filled with blood; 4 = profuse bleeding).
2. MPI (Marginal Plaque Index [24]) and TQHI (Turesky’s modification of the plaque index of Quigely and Hein [25]). The MPI assesses the presence (= 1) or absence (= 0) of plaque adjacent to the gingival margin within eight equal sized sections of a tooth (four at the inner and four at the outer gingival margin, respectively). The TQHI assesses plaque at the entire dental crown at two sites per tooth (inner and outer surface). Each site is given a score of 0 to 5 (0 = no plaque; 1 = several flecks of stain at the cervical margin; 2 = thin continuous band of plaque; up to 1 mm at the cervical margin; 3 = a band of plaque wider than 1 mm but covering less than 1/3 of the crown; 4 = plaque covering at least 1/3 but less than 2/3 of the crown; 5 = plaque covering 2/3 or more of the crown).
3. Clinical probing depth (PD) was recorded with a PCP-UNC 15 probe with a mm scale from 0 to 15 mm.

### Statistics

Statistical analyses were conducted using IBM SPSS statistics version 27.0.

To avoid distortions due to outlying data, persons with outlying values (3 standard deviations from the mean) in behavioural data were excluded from further descriptive analyses. For the remaining participants, all cardinal scaled parameters were first analysed for normality by Kolmogorov–Smirnov test (*p* < 0.05) and visual analysis. If no deviations from the normal distribution were detected, the mean and standard deviation is reported. In all other cases, the median and the 1st and 3rd Quartile (Q1, Q3) or the frequency distributions are depicted as appropriate. In Germany, group and individual prophylaxis measures for oral hygiene education in kindergartens and schools were implemented as a mandatory social legislation only since 1989. Mann Whitney U-Tests (*p* < 0.05) were computed to compare the behaviour of those parents, who were too old to have participated in group and individual prophylaxis measures as children and adolescents (48 years and older) and those who were young enough to having had profited from both (42 years and younger).

## Results

A flow diagram of recruitment is provided in the appendix (Additional file 1: Figure S1). Of the N = 66 parents, seven had at least one outlying value in the behavioural parameters and were thus excluded from further analyses. The appendix (Additional file 1: Table S1) shows the demographic, behavioural and clinical data of these participants.

### General description of the sample

Table 1 shows the demographic and clinical data of the remaining n = 59 parents. Most of them were female, indicating that the mothers were the major responsible for oral hygiene education. The majority of the parents had a university entrance diploma (UED), which is more than the German average (34% [26]). Less than 10% of the parents smoked, which is below the German average of 23–25% in women and 30–36% in men in the age groups of 30–60 years [27]. The mean number of filled teeth was well within the range of representative German samples of younger adults (35–44 years), as were the number of decayed and missing teeth, the number of pontics, and the prevalence of PD ≥ 6 mm. The prevalence of PD ≥ 4 mm was higher than the 35–44 year old German men (63%) and women (55% [27]). More than 50% of the papillae showed positive PBI scores. The mean PBI score indicates that most of the sites showed only mild bleeding (PBI scores 1–2). Positive PBI values were seen more often on the inner surfaces in comparison with the outer surfaces.

**Table 1:** Characteristics of the sample

	*M* ± *SD* [min, max]* n/n
Age	44.6 ± 5.3 [33, 57]
Sex (female/ male/ non-binary)	50/8/1
Educational status (below UED/UED)	13/46
Smoking (non-smoker/smoker)	54/5
*Dental status (including 3rd molars)*	
Healthy teeth	15.2 ± 5.4 [1, 27]
Crowns (0/1–3/4–18)	29/12/18
Pontics (0/1–4)	51/8
*DMFT-Index (without 3rd molars)*	
Number of decayed teeth: 0 /1–3	48/11
Number of missing teeth: 0/1–4/5–7	27/27/5
Number of filled teeth: 0/1–3/4–18	29/12/18
DMFT	12.8 ± 5.0 [1, 23]
*Periodontal status (including 3rd molars)*	
PBI mean score	0.96 ± 0.41 [0.06, 2.64]
PBI% bleeding full mouth	52.4 ± 17.8 [ 6.3, 98.2]
PBI% bleeding outer surfaces	38.9 ± 19.8 [ 0.0, 96.3]
PBI% bleeding inner surfaces	65.9 ± 20.3 [12.5, 100]
Number of teeth with PD ≥ 4 mm: 0/1–2/3–5/6–8/9–13/21	12/19/14/9/4/1
Number of teeth with PD ≥ 6 mm: 0/1/2–6	50/5/4

*Only shown when no deviation from normal distribution is detected

*UED* university entrance diploma; PBI: Papillary Bleeding Index; PD: Probing depth (number of teeth)

### Plaque before and after tooth brushing

**Fig. 1:**
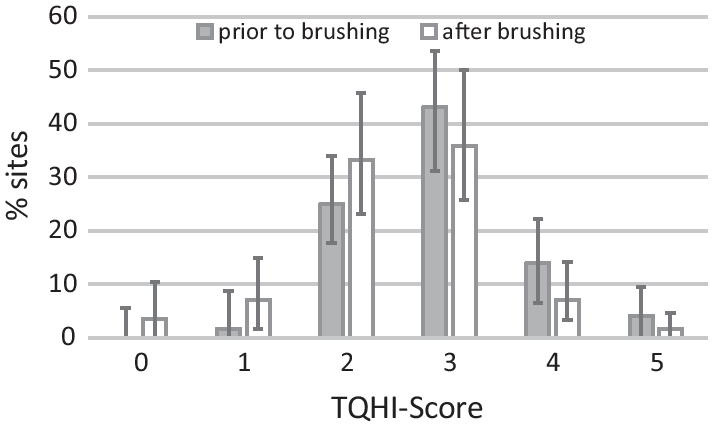
Distribution of TQHI scores prior to and after brushing (median and interquartile differences) in n = 59 parents. Scores 1 and 2 reflect sites at which the plaque is solely situated at the gingival margins, scores 3–5 indicate that plaque is also found in the 1st, 2nd and 3rd third of the crown, respectively. Increasing percentages of sites with score 1 and 2 after brushing indicate that participants manage to remove plaque at more coronal parts of the crown but not at the gingival margin

Table 2 shows the plaque levels immediately prior to and after tooth brushing. Tooth brushing was more effective on the outer surfaces than the inner surfaces. Figure 1 shows the distribution of TQHI-scores prior to and after brushing. While the percentage of sites with TQHI scores 3–5 decreased after brushing, the percentage of sites with scores 1 and 2 increased.

**Table 2:** Plaque levels prior to and after tooth brushing

	Prior to tooth brushing	Immediately after tooth brushing
	*M* ± *SD* [min, max]* Md (Q1; Q3)	*M* ± *SD* [min, max]* Md (Q1; Q3)
*Full mouth*		
TQHI	2.75 ± 0.49 [1.67, 4.12]	2.39 ± 0.50 [0.96, 3.50]
MPI all sections	78.7 ± 12.2 [49.5, 100]	68.4 ± 14.0 [22.9, 97.0]
MPI cervical sections	68.6 ± 16.4 [31.5, 100]	55.4 ± 10.5 [22.8, 91.0]
MPI proximal sections	93.8 (84.8, 98.2)	87.5 (77.7, 95.2)
*Outer surfaces*		
TQHI	2.68 ± 0.56 [1.46, 4.44]	2.22 ± 0.60 [0.57, 3.84]
MPI all sections	71.7 ± 16.0 [37.0, 100]	58.3 ± 16.4 [18.4, 99.0]
*Inner surfaces*		
TQHI	2.81 ± 0.55 [1.48, 4.04]	2.55 ± 0.54 [1.35, 3.74]
MPI all sections	88.0 (77.7, 94.6)	80.4 (69.4, 89.6)

*Only shown when no deviation from normal distribution is detected

*TQHI* Turesky modification of the Plaque Index of Quigley and Hein, *MPI* Marginal Plaque Index

### Tooth brushing behaviour

Table 3 shows the overall tooth brushing behaviour of the parents. The mean tooth contact was more than 1.5 times the recommended duration (97.5 s) taught during group prophylaxis. Nine parents brushed less than 97.5 s. Tooth brushing instructions distribute 46% of the tooth contact time to inner surfaces, 31% to occlusal surfaces and 23% to outer surfaces. The respective percentage in the behaviour of the parents were 27%, 26%, and 47% for inner, occlusal, and outer surfaces.

**Table 3:** Tooth brushing behaviour

	*M* ± *SD* [min, max]* n/n Md (Q1; Q3)
*Tooth contact time (s)*	154.6 ± 57.7 [54.7, 329.3]
Inner surfaces	41.1 ± 23.9 [0.0, 108.5]
Outer surfaces	73.1 ± 32.9 [27.2, 174.2]
Occlusal surfaces	40.4 ± 24.1 [5.8, 116.6]
*% of tooth contact time*	
Inner surfaces	26.7 ± 12.7 [0.0, 51.4]
Outer surfaces	47.2 ± 9.7 [31.0, 73.8]
Occlusal surfaces	26.2 ± 12.0 [5.1, 60.3]
*% of movements on inner surfaces*^a^	
Circular (0%/6%/10–15%/29%)	52/1/4/1
Horizontal	46.0 ± 31.0 [0.0, 100]
Vertical	52.2 ± 30.4 [0.0, 100]
*% of movements on outer surfaces*	
Circular	66.0 ± 24.7 [2.8, 100]
Horizontal	14.4 (1.7, 32.9)
Vertical (0–4%/14–20%/21–40%/46–68%)	39/2/13/5
*% of movements on outer surfaces when jaws closed (“Tiger bite”)*	
Circular	85.4 (57.6, 98.2)
Horizontal	3.7 (0.0, 24.6)
Vertical (0–2%/12–15%/21–40%/31–54%)	44/4/7/4
*% of movements on outer surfaces when jaws open*	
Circular	35.1 (14.2, 77.7)
Horizontal	29.4 (7.5, 72.7)
Vertical (0–6%/14–20%/21–34%/51–92%)	39/2/7/11

*Only shown when no deviation from normal distribution is detected

^a^Not including that Person, who did not brush inner surfaces at all

**Fig. 2:**
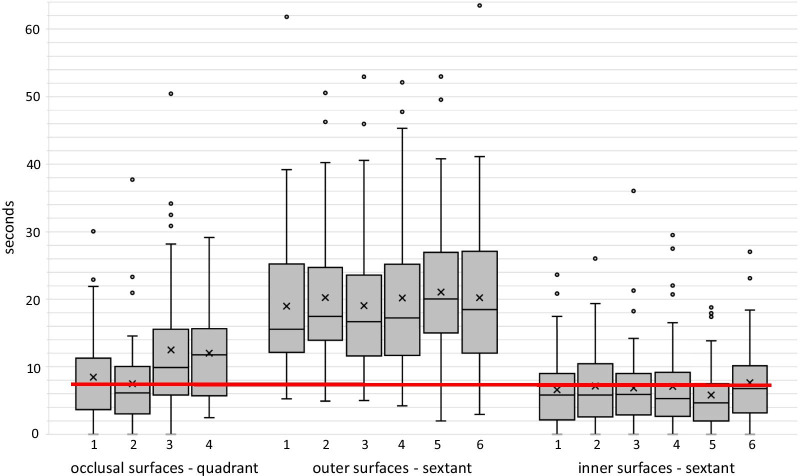
Box plots of the duration of tooth contact on occlusal surfaces (quadrant 1–4) and on outer surfaces (sextant 1–6) and inner surfaces (sextant 1–6) in N = 59 parents. The line within a box indicates the median, the X the mean value. The upper and lower borders of a box represent the 25 and 75% quartiles. The whiskers represent the highest and lowest values observed within the borders for outlying values (> 1.5 times the box length [i.e., interquartile difference] above and below the upper and lower quartiles, respectively), and the symbols reflect extreme values. Children are taught to brush each quadrant and each sextant for 7.5 s (red line) with their jaws closed when brushing outer surfaces (and thus counting the time for two antagonistic sextants at a time). Accordingly, in this graph tooth contact while brushing with mandibles closed is attributed to both antagonistic sextants in order to match this

**Fig. 3:**
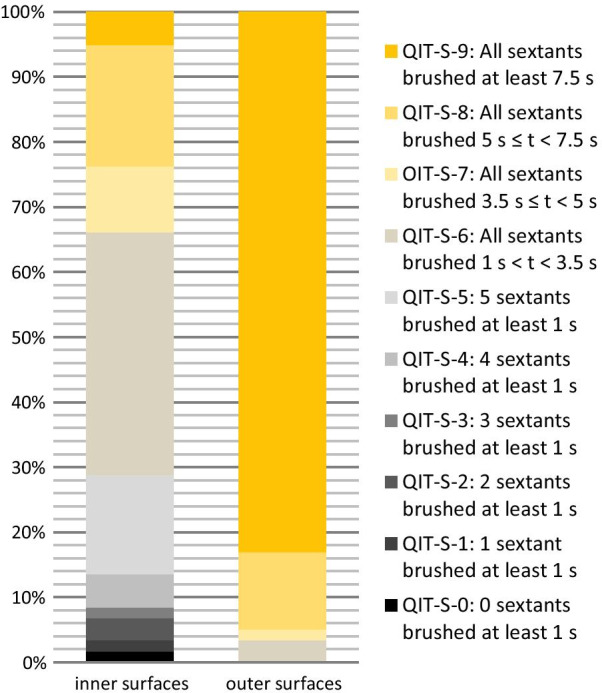
QIT-S scores at inner and outer surfaces. QIT-S scores below 6 indicate neglect of at least one sextant in N = 59 parents

Figure 2 shows in detail how parents distributed tooth contact across the quadrants and inner and outer sextants. Figure 3 depicts the QIT-S for inner and outer surfaces. Seventeen parents (29%) neglected at least one sextant when brushing the inner surfaces and 3 (5%) brushed all sextants for at least 7.5 s. When brushing outer surfaces parents did not neglect any sextants and 49 (83%) brushed all outer sextants by at least 7.5 s.

**Fig. 4:**
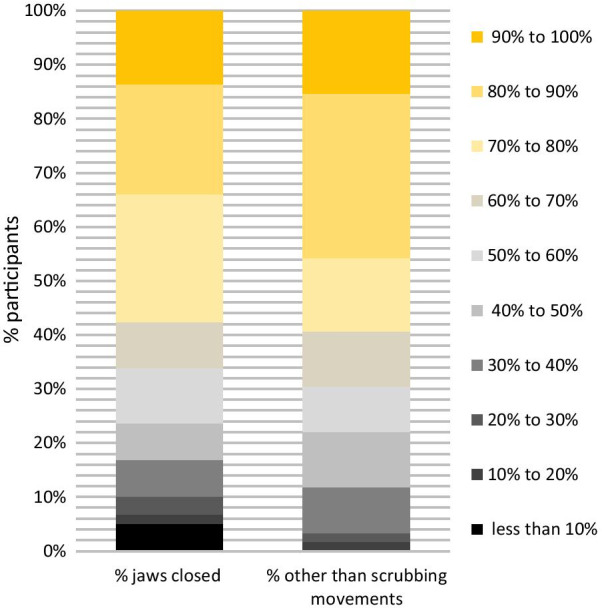
Percentage of time by which parents closed their jaws while brushing outer surfaces (left column) and percentage of time they brushed their lateral surfaces by other than scrubbing movements in N = 59 parents

Regarding tooth brushing movements, children are instructed to brush their inner surfaces with vertical movements and their outer surfaces with circular movements. In parents, vertical movements predominated on the inner and circular on the outer surfaces (Table 3). However, parents also spent 46% of their brushing time on the inner surfaces with horizontal movements. Horizontal brushing at outer surfaces was less common, especially during those periods when parents closed the jaws while brushing (Table 3). Figure 4 shows to which extent parents kept their jaws closed while brushing outer surfaces.

While circular movements on the inner surfaces were rarely shown, 18 parents applied vertical movements when brushing the outer surfaces by more than 20% of the total brushing time (Table 3). Most of these 18 parents combined vertical and circular movements on the outer surfaces and they showed a tendency to brush sextants 1 and 6 more by vertical movements than the other sextants (see Additional file 1: Figures S2 and S3 in the appendix). Figure 4 shows to which extent parents brushed their lateral surfaces with movements other than scrubbing.

To see whether there is an association between scrubbing and neglect of oral surfaces, Spearman rank correlations were computed. The duration by which parents scrubbed was not related to either of the factors: total time, percentage of tooth contact time they brushed their oral surfaces and the QIT-S for oral surfaces (−0.135 < rho < 0.060; all *p* > 0.310).

Only one parent of the total sample (N = 66) applied the modified Bass technique. This was excluded due to outlying values.

### Comparison of the behaviour of older and younger parents

Parents who were young enough (≤ 42 years) to experience group and individual prophylaxis during childhood and adolescence (n = 21, 2 m, 19f) were compared to those who were 48 years and older and could thus not profit from these measures (n = 16, 3 m, 12f, 1non-binary). Due to violations of the normal distribution assumption, Mann-Whitney U-Tests were computed. These tests failed to reach statistical significance despite not accounting for alpha-error inflation (all *p* > 0.10; see Additional file 1: Table S2 of the appendix).

## Discussion

As parents are responsible for the adoption of proper tooth brushing behaviour of their children in daily life [28–30], the present study aimed to analyse the brushing behaviour of the parents.

Most of the participating parents were female, indicating that the mothers were primarily responsible for oral health education. This fits well with the other studies showing that health education is considered mothers’ rather than fathers’ responsibility [31]. Participants in our study group were better educated and smoked less than the German average [26, 27]. The difference regarding education might result in a better performance in the present sample as compared to the German average. Regarding clinical data, dental and periodontal health was within the range of the representative German samples [5].

Parents’ brushing time exceeded the benchmark (97.5 s) by one minute. The guidelines of the European Federation of Periodontology consider 2 min of brushing to be effective in low risk patients. The high risk patients, however, might need more time [32]. Due to the extent of gingivitis in the current sample, one might consider them as high risk rather than low risk group. In that case, the two and a half minutes of brushing time might have been at the lower edge of what would be necessary. On the other hand, merely extending brushing time beyond that level apparently does not increase the brushing effectiveness considerably [33]. One aspect, which might impede the further increase in effectiveness, is the distribution of brushing time across the surfaces. Indeed, the parents paid lesser time to brush inner in comparison with the outer surfaces. Nearly 30% completely neglected the inner surfaces of at least one sextant (see Fig. 2). One explanation could be that they have an inadequate concept of what constitutes good brushing. Two recent studies support this notion. One of the studies asked a representative sample of German adults an open-ended question regarding the areas in which tooth brushing was of special importance. Less than 10% mentioned inner surfaces [34]. The other study [15] compared brushing behaviour of a cohort of 18-year-olds instructed to brush as usual to that of another cohort who brushed to the best of their abilities. Even though the total brushing time of the latter exceeded that of the first by 50 s, the brushing time of inner surfaces was nearly the same in both groups (26 and 31 s; [15]). Drawing from these studies it appears that the concept of good brushing is to brush visible surfaces more than the other sites. This was also observed in this study. The parents brushed the best visible frontal sextants even longer than the lateral surfaces whilst brushing the outer surfaces (see Fig. 1).

Besides the neglect of inner surfaces, the oral hygiene data strongly indicate that they did not manage to reach the gingival margins while brushing. Despite brushing the outer surfaces for more than a minute, the MPI levels remained high. On 58% of the gingival sections of the outer surfaces, plaque persisted after brushing. This was even worse on the inner surfaces. The levels of remaining plaque after tooth brushing to the best of one’s abilities were comparable to those of earlier studies with adults [7–9, 13]. A closer look at TQHI-Scores supports the notion that they particularly missed the gingival margins. This is revealed by the increasing percentage of sites with scores 1 and 2 (see Fig. 1). These reflect plaque adjacent to the gingival margin and are given only if there is no additional plaque on the more coronal aspects of the crowns (which leads to scores 3–5). The parents obviously managed to brush away parts of this more coronal plaque but had problems to remove plaque close to the gingival margins. A more in depth analysis of the MPI values supports the assumption that this reflects not only deficient proximal hygiene but also the cervical sections of the gingival margin. More than half of those showed persistent plaque after brushing (Table 2). The cervical sections reflect those parts of the gingival margin that cannot be accessed by interdental aids but readily respond to training of a brushing technique [24]. Furthermore, it has been shown that dental professionals could achieve nearly perfect oral cleanliness on the gingival margins by means of manual tooth brushing [35].

One might thus assume that parents applied inappropriate brushing techniques. However, when comparing parents’ behaviour to the brushing techniques their children learn during prevention programs, the results partly contradict this assumption. Circular movements predominated on the outer surfaces and vertical on the inner surfaces. Thus, it appears that parents intended to comply with the recommended brushing movements for inner and outer surfaces. Nevertheless, the horizontal scrubbing was also very common on the inner surfaces. This resembles what has been observed in adolescents and young adults [14, 17]. Perhaps this indicates that the vertical movements on the inner surfaces are uncomfortable and thus people tend to return to scrubbing. This notion warrants further investigation also for another reason. A recent systematic review on brushing techniques identified only four RCTs where adult participants were trained in using a brushing technique [36]. Interestingly, none of them revealed results in favour of a brushing technique involving vertical movements.

Parents examined in this study brushed the inner surfaces longer and neglected lesser sextants than their children or other adolescents observed in previous studies [15–17]. Notwithstanding, their deficits regarding distribution of brushing time resemble those of adolescents, they brush their outer surfaces disproportionally longer and neglect inner surfaces. They do so even in the context of a tooth brushing study when they know that their behaviour is filmed and when they know that they became part of the study because they ascribed themselves the main responsibility for the oral hygiene education of their children. One must thus assume that either they are unaware of the neglect or they do not consider inner surfaces important enough to brush longer. As a result, it would probably be difficult for them to teach their children with sufficient attention to brush their inner surfaces and to serve as good role models in this concern. Regarding the application of brushing techniques, the parents’ behaviour was similar to what their children learn. Nevertheless, the parents preferred using scrubbing movements especially on the inner surfaces in spite of other advice. Thus, with respect to the brushing technique the example they give to their children when they brush their own teeth might be not so much a good role model, as well. Unfortunately, video observation does not allow for a valid estimation of the position of the tooth brush bristles on the gingival margins. The current analysis, however, indicates that parents neglected this area, too. It appears that similar to the brushing of inner surfaces there is insufficient awareness of the meaning of oral cleanliness on the gingival margins. It is therefore unlikely that the parents have the capacity to teach proper oral hygiene with respect to gingival margins to their children. Taken together the results indicate that the parents might have difficulties serving as proper role models for their children with regard to three important aspects: Brushing of inner surfaces, application of the recommended brushing techniques and removal of plaque at the gingival margins.

The study assessed the best possible tooth brushing behaviour of an unselected sample of middle-aged adults. Importantly, a considerable degree of gingivitis and first clinical signs of periodontitis emerge at this age [5]. These indicators of insufficient oral hygiene are found in the present sample as well (see Table 1). In clinical practice, problems regarding oral hygiene often are attributed to insufficient oral health motivation [2]. The data of the present analysis, however, bring another explanation: Obviously, the middle-aged adults examined here also lack the abilities or concepts necessary to remove plaque effectively. Thus, they may need more training. The present research indicates that this training should emphasize sites of brushing, in particular inner surfaces and the areas adjacent to the gingival margin. Interestingly, the tooth brushing behaviour of those parents who were young enough to profit from group and individual prophylaxis when they were children did not differ from those who were too old to take any direct advantage of it. Instead, irrespective of their age parents mimicked what their children learn: Brushing the outer surfaces with jaws closed and with circular movements and the inner surfaces with vertical movements (see Additional file 1: Table S2 of the appendix). One might thus assume that parents re-learn their oral hygiene behaviour with their children. From a psychological point of view, this is the most probable. Daily oral hygiene is not only a health behaviour but also a habit which most people perform every day without deep thoughts about what they are doing [37]. When teaching their children, however, they have to become aware of what they are doing. It is conceivable that they seek advice from the materials provided for parents during group prophylaxis [19, 20]. This would open up an important perspective. Parenthood might be a sensitive stage that makes parents particularly receptive to oral hygiene instructions. To take full advantage of that, training of parents should be actively included into group and individual prophylaxis for their children. This might help to improve both their children’s and their own oral health. As discussed above, however, the focus and content of the training needs to be reconsidered to become more effective.

Although the study was conducted in a diligent and highly standardised way, there are also some limitations. The sample size of the study is small due to the complexity of video analyses, which last 4–8 h per participant per single coding (as demonstrated, additional double codings are necessary to identify assessment problems like an observer drift). Thus, large representative surveys of tooth brushing performance are impossible with the technology currently available. Regarding oral health, the current sample was well within the range of representative studies. However, the individuals were better educated than the average. Thus, the current study rather overestimates than underestimates the general capacity of tooth brushing in parents. Most of the participants of the current study were women. In future studies it is also important to examine fathers’ behaviour. In doing so, one would gain additional insights into male oral hygiene behaviour. The current analysis refers to the tooth brushing behaviour, when the parents were asked to perform to the best of their abilities. Thus, there is no information about brushing behaviour in everyday life. The present data merely illustrate the maximum to be expected and in particular amongst well-educated individuals. Even tooth brushing under these laboratory conditions is insufficient and demands improvement. The current analysis focused on manual tooth brushing. One might argue that powered tooth brushing would lead to better results. However, recent analyses indicate that regular powered tooth brushing is of no advantage compared to manual tooth brushing [14, 38]. The probable reasons for this could be drawn from this study. Brushing the right areas, which seemingly causes the most problems while brushing manually, is not automatically overtaken by an electric toothbrush, whereas the brushing technique overtaken by an electric toothbrush automatically does not seem to be highly problematic when brushing manually.

## Conclusions

The main question of the current study was whether parental tooth brushing performance is appropriate to serve as a role model for children’s tooth brushing performance. The current research indicates that this might not be the case. Parents’ own tooth brushing performance shows important deficits, which should be overcome. Future research needs to identify the most effective way to do so.

## Supplementary Information

**Additional file 1.** Flowchart of recruitment, characteristics of excluded participants, relationship between vertical and circular movements at outer surfaces, distribution of vertical movements across sextants at outer surfaces and details of the comparison between older and younger adults with respect to brushing behaviour.

## Data Availability

The datasets used and/or analyzed during the current study are available from the corresponding author on reasonable request. For privacy reasons, however, individual data allowing for the identification of participants (e.g. videos) cannot be made available.

## Abbreviations

AR: Alexander Ritsert (Author)
COVID: corona virus disease
DB: assistant DB
ICC: intra class correlations
KB: assistant KB
MPI: Marginal Plaque Index
MS: assistant MS
PBI: papillary bleeding Index
PD: clinical probing depth
PH: assistant PH
Q1: 1st quartile
Q3: 3rd quartile
QIT-S: Quality Index of tooth brushing regarding brushing time in sextants
SPSS: statistical package for the social sciences
SE: Stefanie Ebel (Author)
SS: Sadhvi Shankar-Subramanian (Author)
TQHI: Turesky’s modification of the plaque index of Quigley and Hein
TS: assistant TS
WHO: World Health Organization
WP: assistant WP
ZE: Zdenka Eidenhardt (Author)

## Footnotes

1 The limitation to these specific age cohorts results from considerations relevant to the analysis of adolescents’ behaviour and is of no further meaning for the aims of the present analysis. More details regarding the selection of these cohorts can be found in the publication describing the adolescents’ behaviour [17].

## References

[1] Baehni PC. Translating science into action–prevention of periodontal disease at patient level. Periodontology. 2000. 2012;60:162–72. 10.1111/j.1600-0757.2011.00428.x. 10.1111/j.1600-0757.2011.00428.x 22909114

[2] Tonetti MS, Eickholz P, Loos BG, Papapanou P, van der Velden U, Armitage G (2015). Principles in prevention of periodontal diseases: consensus report of group 1 of the 11th European workshop on periodontology on effective prevention of periodontal and peri-implant diseases. J Clin Periodontol.

[3] Petersen PE, Ogawa H. The global burden of periodontal disease: towards integration with chronic disease prevention and control. Periodontology. 2000. 2012;60:15–39. 10.1111/j.1600-0757.2011.00425.x. 10.1111/j.1600-0757.2011.00425.x 22909104

[4] Jin LJ, Lamster IB, Greenspan JS, Pitts NB, Scully C, Warnakulasuriya S (2016). Global burden of oral diseases: emerging concepts, management and interplay with systemic health. Oral Dis.

[5] Jordan AR, Micheelis W (2016). Fünfte Deutsche Mundgesundheitsstudie (DMS V) [fifth german oral health study].

[6] Tonetti MS, Jepsen S, Jin L, Otomo-Corgel J (2017). Impact of the global burden of periodontal diseases on health, nutrition and wellbeing of mankind: a call for global action. J Clin Periodontol.

[7] Harnacke D, Beldoch M, Bohn G-H, Seghaoui O, Hegel N, Deinzer R (2012). Oral and written instruction of oral hygiene: a randomized trial. J Periodontol.

[8] Harnacke D, Mitter S, Lehner M, Munzert J, Deinzer R (2012). Improving oral hygiene skills by computer-based training: a randomized controlled comparison of the modified Bass and the Fones techniques. PLoS ONE.

[9] Deinzer R, Harnacke D, Mengel R, Telzer M, Lotzmann U, Wöstmann B (2016). Effectiveness of computer based training (CBT) on toothbrush skills of patients treated with crowns. A randomized control trial. J Periodontol.

[10] Harnacke D, Stein K, Stein P, Margraf-Stiksrud J, Deinzer R (2016). Training in different brushing techniques in relation to efficacy of oral hygiene in young adults: a randomised controlled trial. J Clin Periodontol.

[11] Weik U, Cordes O, Weber J, Krämer N, Pieper K, Margraf-Stiksrud J, Deinzer R. Toothbrushing performance and oral cleanliness after brushing in 12-year-old children. JDR Clin Transl Res. 2020. 10.1177/2380084420975333. 10.1177/2380084420975333 PMC8674791 33251929

[12] van der Weijden FA, Slot DE (2015). Efficacy of homecare regimens for mechanical plaque removal in managing gingivitis a meta review. J Clin Periodontol.

[13] Ebel S, Blättermann H, Weik U, Margraf-Stiksrud J, Deinzer R (2019). High plaque levels after thorough toothbrushing: what impedes efficacy?. JDR Clin Transl Res.

[14] Petker W, Weik U, Margraf-Stiksrud J, Deinzer R (2019). Oral cleanliness in daily users of powered vs. manual toothbrushes: a cross-sectional study. BMC Oral Health.

[15] Deinzer R, Ebel S, Blättermann H, Weik U, Margraf-Stiksrud J (2018). Toothbrushing: to the best of one’s abilities is possibly not good enough. BMC Oral Health.

[16] Deinzer R, Cordes O, Weber J, Hassebrauck L, Weik U, Krämer N (2019). Toothbrushing behavior in children: an observational study of toothbrushing performance in 12 year olds. BMC Oral Health.

[17] Eidenhardt Z, Ritsert A, Shankar-Subramanian S, Ebel S, Margraf-Stiksrud J, Deinzer R (2021). Tooth brushing performance in adolescents as compared to the best-practice demonstrated in group prophylaxis programs: an observational study. BMC Oral Health.

[18] Ganss C, Duran R, Winterfeld T, Schlueter N (2018). Tooth brushing motion patterns with manual and powered toothbrushes: a randomised video observation study. Clin Oral Investig.

[19] Zahnputz-Zauber. Zahnputz-Zauberlied für die KAI plus Systematik - mit professioneller Anleitung [Toothbrushing Wizard Song for the KAI plus systematics: with professional instruction]. 2012. https://www.youtube.com/watch?v=XhcekPpzP5s. Accessed 31 Mar 2021.

[20] Deutsche Arbeitsgemeinschaft für Jugendzahnpflege [German working group for adolescent dental care]. Wie soll mein Kind sich die Zähne putzen. [How should my child brush his/her teeth.]. 2013. https://www.daj.de/FAQ.9.0.html. Accessed 31 Mar 2021.

[21] WHO (2013). Oral health surveys: basic methods.

[22] Saxer UP, Mühlemann HR (1975). Motivation und Aufklärung. [Motivation and education]. SSO Schweiz Monatsschr Zahnheilkd.

[23] Rateitschak KH, Rateitschak EM, Wolf HF (1989). Parodontologie [periodontology].

[24] Deinzer R, Jahns S, Harnacke D (2014). Establishment of a new marginal plaque index with high sensitivity for changes in oral hygiene. J Periodontol.

[25] Turesky S, Gilmore ND, Glickman I (1970). Reduced plaque formation by the chloromethyl analogue of victamine C. J Periodontol.

[26] DESTATIS. Bildungsstand: Bevölkerung im Alter von 15 Jahren und mehr nach allgemeinen und beruflichen Bildungsabschlüssen nach Jahren. 2020. https://www.destatis.de/DE/Themen/Gesellschaft-Umwelt/Bildung-Forschung-Kultur/Bildungsstand/Tabellen/bildungsabschluss.html. Accessed 6 Mar 2021.

[27] DESTATIS. Gesundheitszustand und -relevantes Verhalten: Rauchgewohnheiten nach Alter und Geschlecht. 2017. https://www.destatis.de/DE/Themen/Gesellschaft-Umwelt/Gesundheit/Gesundheitszustand-Relevantes-Verhalten/Tabellen/liste-rauchverhalten.html. Accessed 6 Mar 2021.

[28] Mattos MG, Fernandez CA, Masterson D, Maia LC, Neves AdA (2019). Is the caregivers’ oral health related to dental caries in children or adolescents? A systematic review. Clin Oral Investig.

[29] Virgo-Milton M, Boak R, Hoare A, Gold L, Waters E, Gussy M (2016). An exploration of the views of Australian mothers on promoting child oral health. Aust Dent J..

[30] Poutanen R, Lahti S, Tolvanen M, Hausen H (2006). Parental influence on children’s oral health-related behavior. Acta Odontol Scand.

[31] Khadri FA, Gopinath VK, Hector MP, Davenport ES (2010). How pre-school children learn to brush their teeth in Sharjah, United Arab Emirates. Int J Paediatr Dent.

[32] European Federation of Periodontology. Guidelines for effective prevention of periodontal diseases. 2017. https://www.efp.org/perioworkshop/workshop-2014/guidelines/Prevention-of-periodontal-diseases-guidance-for-dental-surgeons.pdf. Accessed 31 Mar 2021.

[33] Creeth JE, Gallagher A, Sowinski J, Bowman J, Barrett K, Lowe S (2009). The effect of brushing time and dentifrice on dental plaque removal in vivo. J Dent Hyg.

[34] Deinzer R, Jordan AR. Periodontitis: still more to learn. submitted.

[35] Deinzer R, Schmidt R, Harnacke D, Meyle J, Ziebolz D, Hoffmann T, Wöstmann B (2018). Finding an upper limit of what might be achievable by patients: oral cleanliness in dental professionals after self-performed manual oral hygiene. Clin Oral Investig.

[36] Rajwani AR, Hawes SND, To A, Quaranta A, Rincon Aguilar JC (2020). Effectiveness of manual toothbrushing techniques on plaque and gingivitis: a systematic review. Oral Health Prev Dent.

[37] Zimmer S, Lieding L (2014). Gewohnheiten und Kenntnisse zur Mundhygiene in Deutschland – Ergebnisse einer bevölkerungsrepräsentativen Befragung. Dtsch Zahnärztl Z.

[38] Rosema NAM, Adam R, Grender JM, van der Sluijs E, Supranoto SC, van der Weijden GA (2014). Gingival abrasion and recession in manual and oscillating-rotating power brush users. Int J Dent Hyg.

