# Supplementary material for: Good role models? Tooth brushing capabilities of parents: a video observation study
Source: BMC Oral Health. 2021 Sep 24;21:469. doi: 10.1186/s12903-021-01823-6 (PMC8461594; doi:10.1186/s12903-021-01823-6)
Supplement: Supplementary file 1 — Additional file 1. Flowchart of recruitment, characteristics of excluded participants, relationship between vertical and circular movements at outer surfaces, distribution of vertical movements across sextants at outer surfaces and details of the comparison between older and younger adults with respect to brushing behaviour. [file 12903_2021_1823_MOESM1_ESM.pdf]

Appendix of

Good role models? Tooth brushing capabilities of parents – A video observation study

Renate **Deinzer**, Prof. Dr.; Department of Medicine, Justus-Liebig-University Giessen, Klinikstr. 29, D-35392 Giessen, Germany; renate.deinzer@mp.jlug.de

Sadhvi **Shankar-Subramanian**, M.Sc.; Department of Medicine, Justus-Liebig-University Giessen, Klinikstr. 29, D-35392 Giessen, Germany; Sadhvi.Shankar-Subramanian@dentist.med.uni-giessen.de

Alexander **Ritsert**; Department of Medicine, Justus-Liebig-University Giessen, Klinikstr. 29, D-35392 Giessen, Germany; Alexander.H.Ritsert@dentist.med.uni-giessen.de

Stefanie **Ebel**; Department of Medicine, Justus-Liebig-University Giessen, Klinikstr. 29, D-35392 Giessen, Germany; Stefanie.Ebel@mp.med.uni-giessen.de

Bernd **Wöstmann**, Prof. Dr.; Department of Medicine, Justus-Liebig-University Giessen, Schlangenzahl 14, D-35392 Giessen, Germany; Bernd.Woestmann@dentist.med.uni-giessen.de

Jutta **Margraf-Stiksrud**, Dr.; retired, Marburg, Germany; margrafs@t-online.de

Zdenka **Eidenhardt**; Department of Medicine, Justus-Liebig-University Giessen, Klinikstr. 29, D-35392 Giessen, Germany; Zdenka.Eidenhardt@mp.med.uni-giessen.de

Corresponding Author:

Prof. Dr. Renate Deinzer

Institute of Medical Psychology

Department of Medicine

Justus-Liebig-University Giessen

Klinikstr. 29

D-35392 Giessen

renate.deinzer@mp.jlug.de

Appendix

Appendix of Deinzer, R., Shankar-Subhramanian, S., Ritsert, A., Ebel, S., Wöstmann, B., Margraf-Stiksrud, J., Eidenhardt, Z.: Good role models? Tooth brushing capabilities of parents – A video observation study

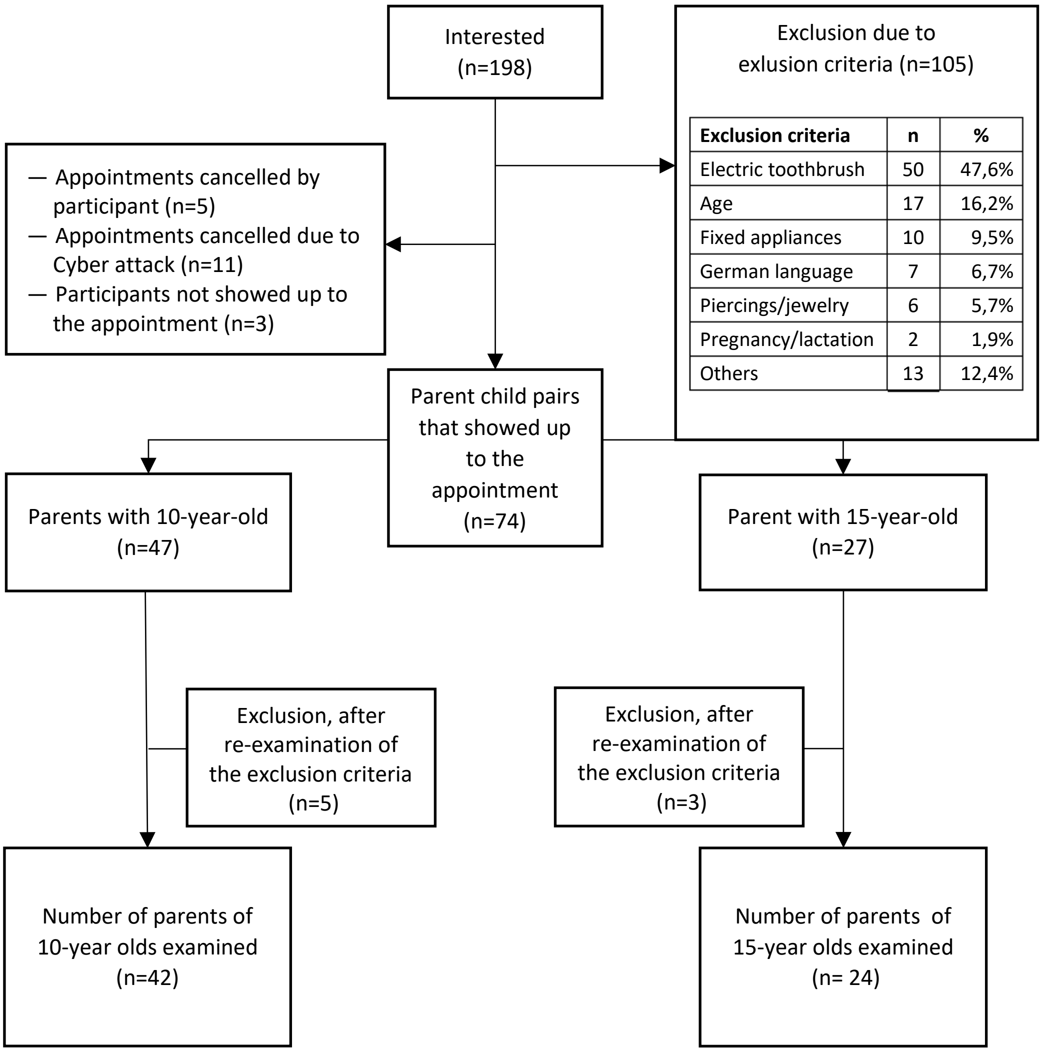

Figure S1: Flowchart of the recruitment (modified from [17]).

Appendix of Deinzer, R., Shankar-Subhramanian, S., Ritsert, A., Ebel, S., Wöstmann, B., Margraf-Stiksrud, J., Eidenhardt, Z.: Good role models? Tooth brushing capabilities of parents – A video observation study

Table S1. Characteristics of the participants excluded due to outlying values in behavioral parameters listed

**demographic data**	A	B	C	D	E	F	G
gender	f	f	f	m	m	m	f
age	50	51	32	41	44	44	42
education	UED	UED	no UED	no UED	no UED	no UED	UED
**behavioral data**							
tooth contact time (seconds)	160.32	204.72	243.00	147.32	85.52	128.68	**403.12**
inner surfaces	73.28	57.28	2.48	27.28	25.84	33.16	**135.44**
outer surfaces	67.60	122.00	64.84	72.48	56.92	75.48	153.76
occlusal surfaces	19.44	25.44	**175.68**	47.56	2.76	20.04	**113.92**
% of tooth contact time							
inner surfaces	45.71	27.98	1.02	18.52	30.22	25.77	33.60
outer surfaces	42.17	59.59	26.68	49.20	66.56	58.66	38.14
occlusal surfaces	12.13	12.43	72.30	32.28	3.23	15.57	28.26
% of movements on inner surfaces							
circular	0.00	0.00	0.00	0.00	0.00	**86.25**	0.00
horizontal	1.15	24.30	0.00	23.75	0.00	10.74	26.61
vertical	98.85	38.48	100.00	76.25	100.00	3.02	73.39
modified Bass technique	0.00	**37.22**	0.00	0.00	0.00	0.00	0.00
% of movements on outer surfaces							
circular	0.00	17.25	16.16	0.00	3.23	62.69	100.00
horizontal	17.10	14.82	45.84	**94.37**	0.70	0.00	0.00
vertical	**82.90**	5.41	38.00	2.65	**96.06**	37.31	0.00
modified Bass technique	0.00	**62.52**	0.00	0.00	0.00	0.00	0.00
% surfaces brushed with jaws closed	55.27	44.16	56.82	91.06	60.30	98.68	98.80
**clinical data**							
DMFT	16	23	17	16	1	13	17
PBI mean score	0.50	1.09	3.39	0.71	0.69	0.55	0.20
PBI % bleeding	26.79	50.00	100.00	44.64	40.32	32.76	11.11
number of teeth with ST >= 4mm	3.00	6.00	19.00	4.00	4.00	21.00	1.00
number of teeth with ST >= 6mm	0.00	0.00	0.00	0.00	0.00	9.00	0.00
MPI all sections prior to brushing	76.68	56.28	100.00	79.46	93.33	93.97	62.50
MPI all sections after brushing	67.86	41.21	96.20	59.38	92.08	78.88	32.41

*bold letters indicate outlying behavioral data

Appendix of Deinzer, R., Shankar-Subhramanian, S., Ritsert, A., Ebel, S., Wöstmann, B., Margraf-Stiksrud, J., Eidenhardt, Z.: Good role models? Tooth brushing capabilities of parents – A video observation study

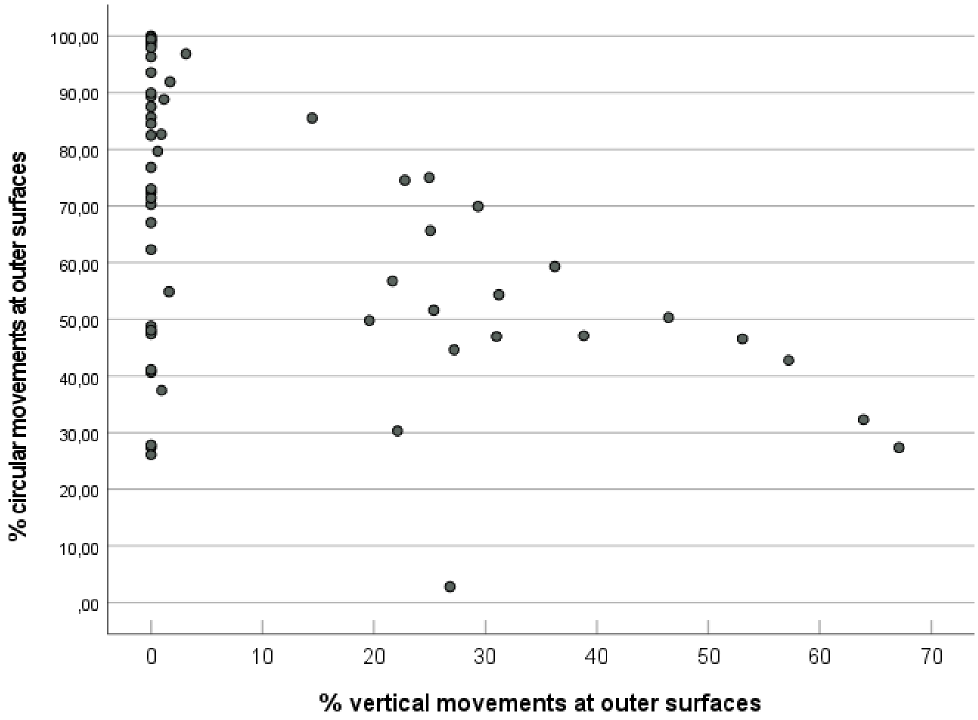

Figure S2: Scatterplot of the percentage of vertical and circular movements at outer surfaces

Appendix of Deinzer, R., Shankar-Subhramanian, S., Ritsert, A., Ebel, S., Wöstmann, B., Margraf-Stiksrud, J., Eidenhardt, Z.: Good role models? Tooth brushing capabilities of parents – A video observation study

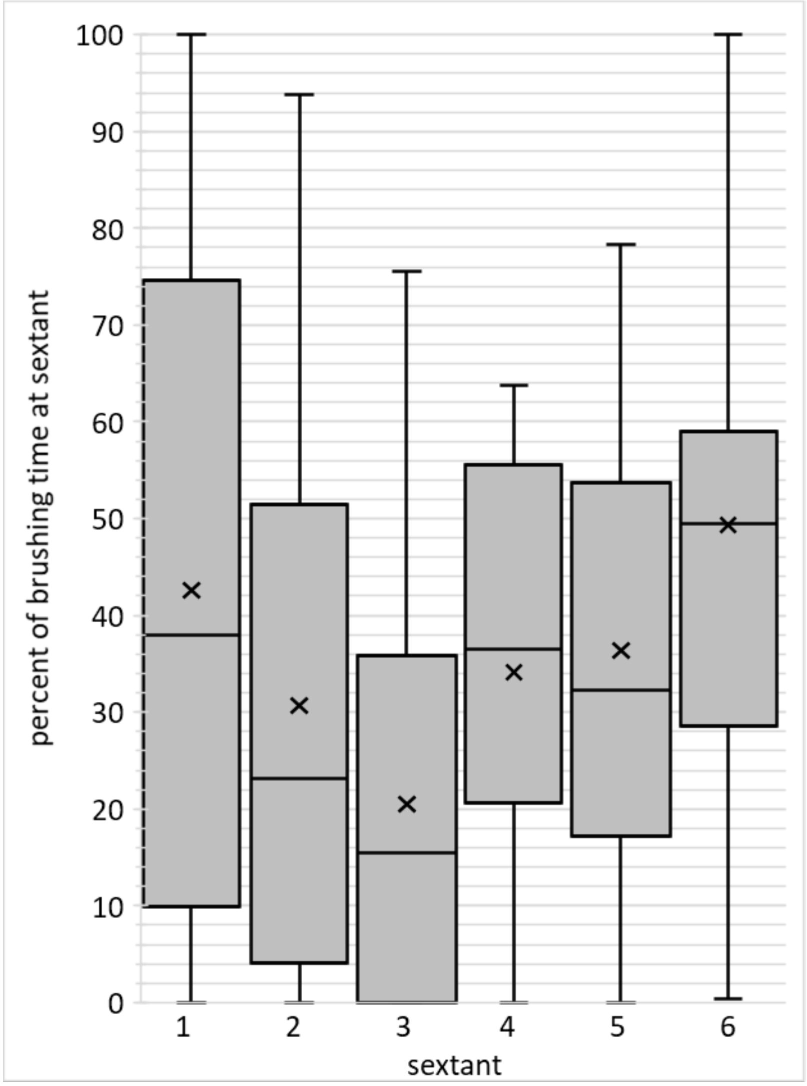

Figure S3: Box Plots of the distribution of vertical movements to sextants at outer surfaces for those n=18 participants who brushed outer surfaces by more than 20% of the time by vertical movements. The line within a box indicates the median, the X the mean value, the upper and lower borders, the 25% and 75% quartiles. The whiskers represent the highest and lowest values observed within the borders for outlying values (>1.5 times the box length [i.e., interquartile difference] above and below the upper and lower quartiles, respectively).

Appendix of Deinzer, R., Shankar-Subhramanian, S., Ritsert, A., Ebel, S., Wöstmann, B., Margraf-Stiksrud, J., Eidenhardt, Z.: Good role models? Tooth brushing capabilities of parents – A video observation study

Table S2: Group differences of younger and older adults (with good or bad chance to profit from group and individual prophylaxis measures as children)

	aged ≤ 42 y (n=21)			Aged ≥ 48 (n=16)			
	1st Quartile	**Median**	3rd Quartile	1st Quartile	**Median**	3rd Quartile	p*
tooth contact time (seconds)	105.16	**135.64**	181.22	109.74	**157.86**	171.29	0.92
inner surfaces	17.74	**25.12**	47.36	27.66	**42.18**	51.96	0.24
outer surfaces	48.48	**60.72**	95.38	55.81	**68.02**	79.94	0.94
occlusal surfces	24.10	**44.04**	62.90	27.24	**37.46**	50.99	0.6
% of tooth contact time							
inner surfaces	16.56	**20.52**	25.00	18.78	**28.82**	40.15	0.11
outer surfaces	43.52	**49.30**	53.96	39.24	**44.75**	50.68	0.2
occlusal surfaces	22.85	**27.81**	35.06	18.26	**24.25**	34.44	0.44
% of movements on inner surfaces							
circular	0.00	**0.00**	0.00	0.00	**0.00**	0.00	0.25
horizontal	21.61	**41.29**	72.09	16.46	**35.29**	45.17	0.46
vertical	25.04	**56.73**	78.39	54.62	**60.00**	76.81	0.58
% of movements on outer surfaces							
circular	49.56	**73.03**	88.72	46.89	**73.47**	91.13	0.80
horizontal	2.24	**14.03**	29.14	1.94	**8.75**	28.74	0.96
vertical	0.00	**0.00**	23.55	0.00	**1.06**	24.51	0.44
% brushing outer surfaces with closed jaws	64.99	**74.72**	88.47	32.74	**76.19**	93.64	0.6
QIT-S							
inner surfaces	5.00	**6.00**	6.00	4.00	**6.00**	7.00	0.52
outer surfaces	9.00	**9.00**	9.00	9.00	**9.00**	9.00	0.45

*Mann-Whithney U Test, exact p
